# Integrated Neurobehavioral and Pharmacological Treatment in Adults with Autism Spectrum Disorder and Bipolar Disorder: A Multiple Baseline Study

**DOI:** 10.1192/j.eurpsy.2026.11184

**Published:** 2026-07-31

**Authors:** N. Varrucciu, R. Di Sarro, A. Di Santantonio, V. Pascale

**Affiliations:** 1 Mental Health, AUSL Bologna, Bologna; 2 NDD, abafordisability, Salerno, Italy

## Abstract

**Introduction:**

Adults with Autism Spectrum Disorder (ASD) and comorbid bipolar disorder present highly complex clinical profiles requiring coordinated neuropsychiatric and behavioral interventions. While pharmacological treatment is essential for stabilizing mood fluctuations and managing acute episodes, medication alone is often insufficient to ensure the acquisition and maintenance of functional independence.

**Objectives:**

This study aimed to evaluate the combined effects of pharmacological management and neurobehavioral treatment, grounded in Applied Behavior Analysis (ABA) and neuropsychological principles, on functional autonomy, mood regulation, and quality of life in adults with ASD and bipolar disorder.

**Methods:**

A multiple baseline design across five adult participants was employed. Individualized pharmacotherapy stabilized mood symptoms, while structured behavioral interventions targeted Activities of Daily Living (ADL) and Instrumental Activities of Daily Living (IADL), including medication adherence, financial planning, and community participation. Behavioral procedures (task analysis, chaining, differential reinforcement) were complemented by cognitive enhancement strategies to strengthen executive functioning, planning, and self-regulation. Outcome measures included direct observation of functional skills, standardized neuropsychological assessments, and validated scales of quality of life and well-being.

**Results:**

The integration of pharmacological treatment with neurobehavioral intervention produced consistent and sustained improvements in all participants. Notable gains were observed in adaptive functioning, inhibitory control, and engagement in structured activities, accompanied by a reduction in the frequency and intensity of problem behaviors. Improvements in autonomy, emotional regulation, and daily functioning were maintained at follow-up, underscoring the durability of the intervention.

**Conclusions:**

Findings indicate that adults with ASD and bipolar disorder benefit most from integrated care models that simultaneously address psychiatric stabilization and functional competence. This comprehensive approach enhances quality of life, well-being, and long-term independence, supporting the value of interdisciplinary treatment strategies in complex clinical populations

**Disclosure of Interest:**

None Declared

